# The role of artificial intelligence in general, and large language models specifically, for understanding addictive behaviors

**DOI:** 10.1111/nyas.15337

**Published:** 2025-04-29

**Authors:** Christian Montag, Haibo Yang, Anise M. S. Wu, Raian Ali, Jon D. Elhai

**Affiliations:** ^1^ Department of Molecular Psychology, Institute of Psychology and Education Ulm University Ulm Germany; ^2^ Academy of Psychology and Behavior Tianjin Normal University Tianjin China; ^3^ Department of Psychology, Faculty of Social Sciences University of Macau Macao China; ^4^ Center for Cognitive and Brain Sciences, Institute of Collaborative Innovation University of Macau Macao China; ^5^ College of Science and Engineering Hamad Bin Khalifa University Doha Qatar; ^6^ Department of Psychology University of Toledo Toledo Ohio USA; ^7^ Department of Psychiatry University of Toledo Toledo Ohio USA

**Keywords:** addiction, Artificial Intelligence Act, Digital Services Act, internet use disorders, large language models

## Abstract

Artificial intelligence (AI) presents a general‐purpose technology built into diverse products including language assistants on smartphones, recommender systems in e‐commerce and social media, and applications in social and industrial robotics. AI became a globally discussed topic when the large language model ChatGPT was launched in November 2022. In the aftermath, scientists in the field of (online) addictive behaviors and internet use disorders have discussed which features or modalities of AI systems underlying video games or social media platforms might result in adverse consequences for users. Therefore, the present short communication sheds light on recent discussions in the realm of addictive behaviors on the eve of the coming AI wave. Furthermore, terms such as ChatGPT addiction are critically discussed and we not only theoretically explain how different AI modalities interact with governing regulation bodies such as the EU with their AI Act, but also personal/psychological factors, paving the way to unique perceived immersive design levels.

## Graphical abstract

The advent of generative artificial intelligence (AI), such as ChatGPT, has led to widespread discussions of how AI will shape individuals and societies. Furthermore, we see that AI is integrated into a growing number of products. For instance, it is well known that social media companies use AI in their recommendation engines. Therefore, the paper discusses how the use of AI in digital products might trigger addictive behaviors.

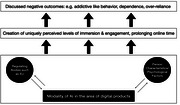

## ARTIFICIAL INTELLIGENCE AND ADDICTIVE BEHAVIORS

Artificial intelligence (AI) represents a key technology impacting societies around the world at the time of this writing.^1^, ^2^, ^3^ AI itself represents a blurry concept because AI technology—coming in different forms—is already embedded in many services people use in everyday life. A classic definition of AI goes back to the Dartmouth Summer Research Project in 1956, where it was put forward that “every aspect of learning or any other feature of intelligence can in principle be so precisely described that a machine can be made to simulate it.”^4^

Today, among AI‐empowered products people are using, the most common are language assistants on smartphones but also newsfeeds within social media apps or highly immersive video game environments. Both social media and video games represent good examples of AI technology being relevant for shedding light on behavioral addictions.^5^ This phenomenon must be seen in a larger context: Although social media addiction or social network use disorder does not represent an official diagnosis yet, researchers have discussed if excessive use of social media can be characterized using an addiction framework.^6^ Recently, large language models (LLMs) and conversational AI agents, exemplified by ChatGPT (for discussion of versions, see below), have been suggested to facilitate addictive patterns of use and attachment among users.^7^ The authors identified four contributing factors: personal relevance as a motivator, parasocial bonds enhancing dependency, productivity boosts providing gratification and fueling commitment, and over‐reliance on AI for decision‐making.

Although it is still too early to settle this discussion about the addictive nature of excessive social media use (or more recently of excessive ChatGPT or LLM use), it has been put forward several times that the data business model operating behind social media companies contributes to addictive‐like behaviors in the context of social media.^8^, ^9^ In short, the industry aims to prolong the online behavior of their users and the quantity of content and interactions they produce, by so‐called persuasive design. While technology design can contribute to triggering addictive and problematic behaviors,^10^, ^11^ it can also be part of the solution.^12^ However, mechanisms to reduce problematic attachments remain uncommon on many widely used social media platforms. Research has proposed sociotechnical solutions that can be integrated into the design of digital media for managing the fear of missing out^13^ and reducing procrastination.^14^

Speaking in terms borrowed from behavioral economics,^15^ users are nudged via platform design to become lured into using an online platform by mechanisms such as push notifications eliciting fear of missing out when not on the platform.^16^, ^17^ When spending time on the platform, design mechanisms such as “likes”, stories, and endless scrolling features aim to prolong the online session and/or foster more engagement (for a general taxonomy of design elements, see Ref. 18). But back to the personalization issue in social media: A critical design feature aiming to prolong online time relies on AI technology, namely, presenting users with personalized content. Social media platforms use such an approach with personalized news in social media feeds to prevent boredom on the platforms. In order to pick the most fitting news for each user, AI is used to study user histories to develop the potentially most relevant and interesting feed, or what Facebook called at the beginning of their personalized newsfeed, the “New York Times of You” (p. 261).^19^ We are left with the question: If AI has been effectively used to gauge user interest and promote prolonged usage and engagement, why cannot it also be used to predict problematic use patterns and recommend ways to keep usage under control?

Researchers have yet to provide solid evidence on the extent to which AI‐empowered design features drive addictive behaviors. Unfortunately, it is difficult to study such research questions because the application programming interfaces (APIs) are closed on many platforms, forbidding examination of the correlation between objectively collected data about interaction patterns of a user with his/her self‐reported addictive tendencies toward social media. Researchers speak in this context of the APIcalypse.^20^, ^21^ Recent published research investigating data from Facebook before the Cambridge Analytica scandal (after which the APIs were closed) demonstrated that “like” metrics are indeed associated with self‐reported addictive‐like behavior.^22^ And the “likes” information is something without doubt being studied by AI to present social media users with the most interesting content.

Social media products are not the only products profiting from AI technology to hold people on virtual platforms. In particular, the gaming industry relies upon AI not only to design immersive online environments but also to allow users to play against artificially intelligent opponents. Furthermore, personalization in collaborative games has been shown to improve engagement in video game playing.^23^ This said, AI and machine learning, which can be seen as an essential AI technique, have also been used to improve “serious games” or educational games.^24^ Therefore, there will be a fine line to walk between where AI simply fosters engagement of their users—in this case, AI becoming more successful with learning—and where engagement results in addictive‐like behaviors toward a certain digital product. Similar thoughts have been put forward when investigating high versus pathological involvements in video games.^25^ Such a distinction is important in order to not over‐pathologize everyday life behavior.^26^ In line with work on different involvement levels with video games,^25^ recent empirical/theoretical observations underline the importance of studying functional impairment to understand addictive‐like behavior.^27^, ^28^ While the need to introduce countermeasures for addictive behaviors, including those driven by AI, is still a topic of debate due to the questionable consideration of AI‐addictive behavior, even in online gambling—a well‐recognized form of internet use disorder—only basic features to prevent addiction and promote controlled usage are typically available, for example, limit setting and helplines. This is despite literature demonstrating the effectiveness of solutions in that domain like breaking user dissociation and immersion through cognitive tasks and informative messages^29^ and psychologically inoculating players by explaining persuasive design elements to enhance critical thinking.^30^

AI needs to be seen as a critical technology being increasingly used in the design and operations of digital products. Therefore, it is not a surprise that, in other areas of addictive behaviors such as gambling disorder, pornography use disorder, or shopping/buying disorder, AI will also play an important role. In general, AI helps the industry to make sense of the large available user data being left behind when spending time on platforms to enhance user experience. At first sight, this can also be positive to lessen the cognitive burden of users being presented with tons of irrelevant information (cognitive overload). But on the dark side, AI‐empowered design can nudge users toward longer online use and likely larger monetary expenses, which then can result for some users in adverse consequences.

## THE RISE OF LLMS AND ADDICTIVE BEHAVIORS

In November 2022, OpenAI launched the AI‐powered LLM ChatGPT, which led to tremendous media coverage around the world and to heated discussion among scientists. To be precise, when ChatGPT 3.5 was launched, it was already characterized by a large number of parameters (an estimate of about 175 billion parameters in its deep neural network) allowing sophisticated human–computer interactions that might form the basis of being perceived as addictive for some users or to form parasocial relationships.^a^ The latest version of OpenAI's LLM “o1” might be even better at this and other tasks (for instance, for “hallucinating” less and being more accurate).

We expect that OpenAI's GPT products will develop toward a more immersive experience over the evolution of their different versions, hence likely exerting a stronger influence on people. But this expectation is difficult to test in hindsight, as the earliest models are not on the market anymore. Moreover, it might be the case that perceiving more restrictions when interacting with later versions of the LLM (due to built‐in safeguards preventing the human–computer interaction from going rogue) might also have an influence on how addictive the product is perceived. So perhaps we see deviations from the assumption that, with every new version of (Chat)GPT being launched, we will see more addictive potential as a result. Of note, when we speak of ChatGPT in this article, we refer to the products being launched to the public (starting with ChatGPT 3.5) and we do this because most readers will immediately know what we are referencing (although OpenAI speaks in their recent models of GPT‐4o or o1 and removed the term “Chat”). Moreover, we use ChatGPT as an example of the family of conversational AIs offering the benefits of adaptability, personalization, context awareness, continuous learning and multimodal functionality, reasoning and problem‐solving, scalability, and real‐time collaboration.^35^

In line with this, we see that, beyond ChatGPT, other LLMs can also be used to query a text‐based AI system to write essays, answer questions, run statistical analyses, and write code. Further, text input can also be directly used to create pictures via products such as DALL‐E2 (https://openai.com/index/dall‐e‐2/) or Midjourney (https://www.midjourney.com). Another prominent example is character.ai, where users can create characters they want to interact with. Such characters can even be celebrities, where people perhaps even form more parasocial relationships. Again, this is something worth being tested.

The discussion around LLMs (and the more general product category of generative AIs) ranges from LLMs hallucinating when providing answers^36^ (see above) to more classic research questions we know from the past when other technologies were introduced. From books to smartphones, it has been described that societies respond with moral panic to new technology in a paper by Amy Orben:^37^ By counteracting the new technology with large concerns (today easily shared by the mass media), stakeholders aim to achieve very different results. This can include concerns to protect users from the real harm of the newly introduced technology to the selfish goals of an agent. This concern could also include a politician showing that he/she is acting in a responsible way, though it might be a strategy to be (re‐)elected. We do not question the real worries and concerns of scientists and politicians and the need to carry out sound research to answer how technology impacts societies, but we also hint at the complex nature of people's reactions to new technology throughout the history of mankind. Again, see for deeper discussions the work by Amy Orben.^37^

Against the background of these historical observations, we mention that early papers have been published studying what has been called “ChatGPT addiction.” In other words, an addiction framework was applied to understand if people show addictive‐like behaviors toward ChatGPT (but as mentioned, more fine, granular discussions around ChatGPT versions or other LLMs are necessary). For instance, a recent case report highlighted that creating a parasocial relationship with a created character on ChatGPT could elicit addictive tendencies in a male patient.^38^ Other researchers put forward a scale to assess problematic ChatGPT use,^39^ by drawing parallels with internet gaming disorder symptoms—such as conflict, loss of control, and mood modification—as a conceptual framework, which raises questions about whether this test reuse‐based approach would address the specific nuances of ChatGPT addiction and over‐reliance on LLMs.^7^ In further new work, scientists speak of ChatGPT dependence,^40^ which was associated with inert thinking in the context of the established I‐PACE theoretical model from behavioral addiction research.^41^

At this early point, it is clearly not easy to characterize (excessive) human behavior in the context of LLM usage, and the cited studies also mentioned the advantages of using this technology. That said, we believe that a single focus on addictive behaviors for describing excessive ChatGPT or LLM use very likely represents an overly narrow path when we really want to understand human–LLM interaction. Therefore, we are convinced of the importance of also investigating other theoretical concepts when trying to understand people's use of ChatGPT or related LLMs such as Llama from Meta or the aforementioned character.ai platform. Aside from testing alternative theories from psychology/psychiatry (such as compulsive behavior), it will be important to also understand in more nonclinical terms what the technology use is doing to people. Here, we prefer the term over‐reliance on technology,^42^ hence following the question of what happens to human thinking when we (constantly) outsource cognitive tasks to machines. Will this outsourcing impact the human mind and our cognitive functions and to what degree? In a way, this resembles the discussion around the smartphone, which has not been settled to this day.^43^ And we should not forget that technology such as AI can uplift people in their work and private lives, leading to what has been coined Co‐Intelligence.^44^

## CONSIDERING THE IMPACT FRAMEWORK IN THE STUDY OF AI WELL‐BEING AND ADDICTIVE BEHAVIORS

As introduced in the beginning, AI represents a blurry concept, and to truly grasp its influence on society and individuals, several categories need to be considered. In this realm, the IMPACT framework might be helpful in describing the Interplay of Modality, Person, Area, Country, and Transparency variables to understand AI's impact.^45^ This framework originally aimed at understanding how AI influences “AI well‐being,” hence our well‐being when we interact directly with AI technology or our well‐being in societies being shaped by AI. The IMPACT framework makes the point that, to understand AI's influence, we have to consider the Modality of the AI system. Is the AI interactive, such as a GPT‐4o? Does it have an output system such as the newsfeed on a social media platform? Can it “see” via computer vision or “read” text? We propose that the mode of operation of an AI system is studied when we think of Modality. The Person variable of the IMPACT framework comprises both sociodemographic and psychological factors. Here, it is investigated to what extent age, gender, education, and personality traits or motives to use AI influence our well‐being. It would not be surprising to observe that these factors play a role, because it has been already shown earlier in other related areas that such factors matter.^46^, ^47^ And motives might also be relevant for an understanding of “ChatGPT addiction” (just used as a working term): The mentioned case study reported that a lonely person used ChatGPT to create someone to have conversations with. Such pseudo‐bonding and parasocial relationships are argued to be unique to ChatGPT and conversational AI agents, distinguishing dependency or addiction to these from typical social media.^7^ This, together with personalized answers and productivity boost, and the associated gratification, might have led to the attachment to and over‐reliance on this medium and addictive‐like behavior.^7^, ^38^ Hence, the use of ChatGPT clearly went hand‐in‐hand with the motive to overcome loneliness, and perhaps also escapist tendencies played a role resulting in unhealthy behavior.

Aside from the Modality and Person categories of the IMPACT framework, the Area and Country categories also need to be mentioned. The Area category describes that the impact of AI on society and AI users depends on the field (area) where AI is applied. One could imagine that if AI technology aims simply at prolonging time on social media or within video games, its influence might be negative. If AI technology is used to counteract unhealthy usage patterns, for example, via mobile sensing or digital phenotyping strategies, it might be positive.^48^ We could imagine that after a certain amount of use time, the AI could recommend engaging in a different activity,^49^ but will such an intervention be sufficient? The AI might devise something cleverer to help users to reduce excessive online time. The relationship between the Person and the Area of application could also be a factor. For example, someone with limited ability to perform job‐related tasks in a specific area may become more attached to ChatGPT as a companion to complement their skills and address shortcomings. In such a case, the concept of Co‐Intelligence may lead to over‐reliance and dependency, resulting in reduced motivation for learning and diminished capacity for independent decision‐making.^7^

The Country/Culture category from the IMPACT framework means that AI's influence will be also shaped by both country and culture variables. Whereas countries and/or regions of the world differ in their attempts to regulate AI technology, cultural specifics such as individualism versus collectivism or individual differences in power distance^50^ might also be relevant to understanding individual differences in the response to an AI‐system (as has been discussed in the context of problematic internet use^51^). Of note, a prominent attempt to regulate AI (to foster trustworthy AI) is represented by the Artificial Intelligence Act (AIA) by the EU. But as mentioned, different regions in the world respond differently to the challenges around AI, for example, Singapore promotes lifelong learning by paying for AI education of their citizens aged 40 or above.

Finally, the “T” of the IMPACT model stands for the Transparency variable. Here, the question is asked: to what degree can the opaqueness of an AI system be reduced via explainable AI (XAI)? For instance, the AI system could devise explanations regarding why it presents users with a certain output. Transparency will be important (also as a Modality) to protect users from AI algorithms creating an overly immersive experience. Transparency can also serve as a countermeasure for addictive behavior, but it should go beyond explaining the algorithm to include how users may be persuaded and how their decision‐making processes might be influenced to foster more healthy behavior.^29^ Recent research has shown an association between belief in conspiracy theories and negative attitudes toward AI, highlighting the importance of expanding transparency to cover aspects such as the data business model and the intentions behind the AI system.^52^

**FIGURE 1:**
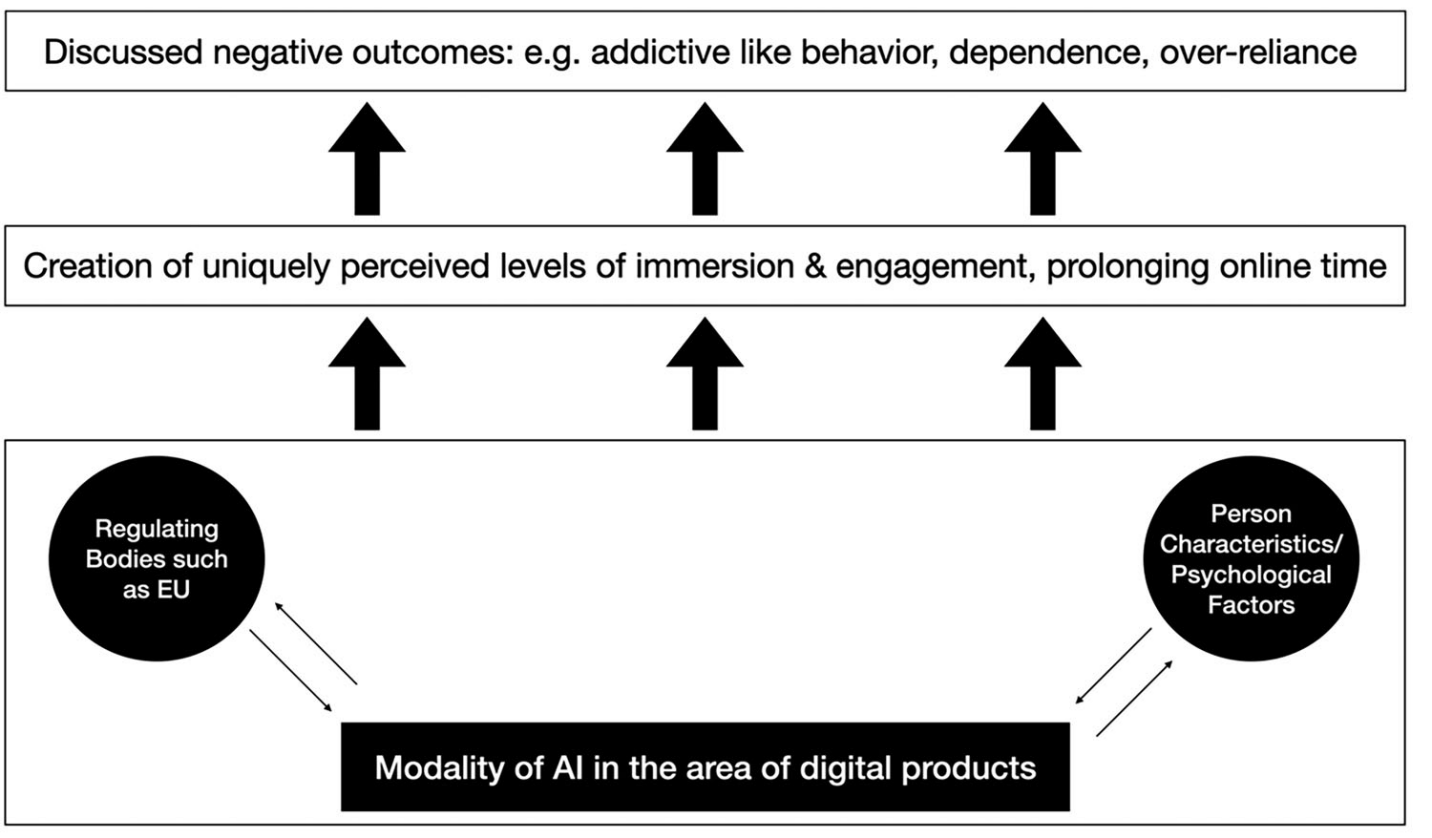
How perceptions and actual consequences of the modality of an AI system might be shaped and lead to adverse outcomes for some users.

In this context, the recent initiative around the Digital Services Act (DSA) by the EU is also noteworthy, aiming to protect minors from harm on so‐called VLOPs (very large online platforms). The DSA speaks in preamble 81 of risks arising “in relation to the design of online interfaces which intentionally or unintentionally exploit the weaknesses and inexperience of minors or which may cause addictive behavior.”^b^ Also, the AIA added paragraph 5(1)(a) prohibiting AI systems deploying “subliminal techniques beyond a person's consciousness or purposefully manipulative or deceptive techniques” resulting in harm (please see^c^ for the full text of the AIA act). In other words, both the surface of platforms regarding their platform design and the underlying algorithms are targets of regulation in the EU, likely shaping the operating AI platforms (and their addictive potential). Therefore, the development, deployment, and operation of AI systems interact both with regulatory bodies and personal/psychological characteristics, explaining the perceived and actual unique level of immersion or prolonged online time on such a platform, paving the way for some individuals toward adverse outcomes such as addictive‐like behaviors or over‐reliance (see Figure 1).

## CONCLUSIONS

The AI wave is hitting societies around the globe. With this, special challenges also arise regarding digital products we use on a daily basis, which may become too immersive or too time‐consuming without proper regulation. The EU Commission put forward prominent regulation schemes with the DSA and AIA, but it is not clear which regulatory approach will be most successful in protecting users of AI systems while not stifling innovation. The unique perceived level of immersion, engagement, or prolonged online time because of an AI system needs to be placed in not only the context of the regulatory environment, but also the personal/psychological characteristics of the user. This of course is still a limited view of what drives human behavior, but names two prominent categories interacting with AI modalities.

## AUTHOR CONTRIBUTIONS

C.M. drafted the first version of the paper, which was critically revised by H.Y., A.M.S.W., R.A., and J.D.E.

## COMPETING INTERESTS

The authors declare no competing interests.

## Data Availability

Data sharing is not applicable to this article as no datasets were generated or analyzed during the current study.

## Footnotes

a Research on the formation of parasocial relationships^31^ and human–computer interaction provides insights into how humans form connections with nonsentient and artificial agents. Individuals may perceive AI chatbots and social robots as social entities capable of understanding, leading to parasocial bonds.^32^, ^33^ ChatGPT and conversational LLMs can be tailored to personalize communication, such as using a friendly tone, avoiding harsh language, or engaging in casual interactions like uploading a photo and asking, “How do I look today?”. This “on‐demand” partnership and sense of assurance may evolve into a form of emotional bonding. Furthermore, ChatGPT can develop into a cognitive partner, fostering intellectual and cognitive dimensions of connection. ChatGPT has redefined AI by becoming more than a task automation tool, positioning itself as a cognitive partner that collaborates with users on giving opinions, not only information. This aligns with Ackoff's DIKW Pyramid, which illustrates the progression from data to wisdom.^34^ Unlike traditional utility‐driven AI, ChatGPT surpasses basic automation by serving as an engaged collaborator, providing support even in scenarios where explicit logic and reasoning chains are not required. In other words, trust in ChatGPT's “wisdom,” derived from vast data and conversational capabilities, may outweigh the demand for explainability, which is a form of parasocial relationship.

b
https://www.eu‐digital‐services‐act.com/Digital_Services_Act_Preamble_81_to_90.html

c
https://artificialintelligenceact.eu/the‐act/
